# Genome-Wide Analysis of the Banana WRKY Transcription Factor Gene Family Closely Related to Fruit Ripening and Stress

**DOI:** 10.3390/plants11050662

**Published:** 2022-02-28

**Authors:** Caihong Jia, Zhuo Wang, Jingyi Wang, Hongxia Miao, Jianbin Zhang, Biyu Xu, Juhua Liu, Zhiqiang Jin, Jihong Liu

**Affiliations:** 1 Key Laboratory of Horticultural Plant Biology, College of Horticulture and Forestry Sciences, Huazhong Agricultural University, Wuhan 430070, China; jiacaihong@itbb.org.cn; 2 Key Laboratory of Tropical Crop Biotechnology, Ministry of Agriculture, Institute of Tropical Bioscience and Biotechnology, Chinese Academy of Tropical Agricultural Sciences, Haikou 571101, China; wangzhuo@itbb.org.cn (Z.W.); wangjingyi@itbb.org.cn (J.W.); miaohongxia@itbb.org.cn (H.M.); zhangjianbin@itbb.org.cn (J.Z.); xubiyu@itbb.org.cn (B.X.); 3 Hainan Key Laboratory for Protection and Utilization of Tropical Bioresource, Hainan Academy of Tropical Agricultural Resource, Chinese Academy of Tropical Agricultural Sciences, Haikou 571101, China

**Keywords:** WRKY, banana, stress, expression analysis

## Abstract

WRKY transcription factors (TFs) play an important role in plant responses to biotic and abiotic stress as well as in plant growth and development. In the present study, bioinformatics methods were used to identify members of the WRKY transcription factor family in the *Musa acuminata* (DH-Pahang) genome (version 2). A total of 164 *MaWRKYs* were identified and phylogenetic analysis showed that MaWRKYs could be categorized into three subfamilies. Overall, the 162 *MaWRKYs* were distributed on 11 chromosomes, and 2 genes were not located on the chromosome. There were 31 collinear genes from segmental duplication and 7 pairs of genes from tandem duplication. RNA-sequencing was used to analyze the expression profiles of *MaWRKYs* in different fruit development, ripening stages, under various abiotic and biotic stressors. Most of the *MaWRKYs* showed a variety of expression patterns in the banana fruit development and ripening stages. Some *MaWRKYs* responded to abiotic stress, such as low temperature, drought, and salt stress. Most differentially expressed *MaWRKYs* were downregulated during banana’s response to Foc TR4 infection, which plays an important role in physiological regulation to stress. Our findings indicate that *MaWRKY21* directly binds to the W-box of the *MaICS* promoter to decrease *MaICS* transcription and then reduce the enzyme activity. These studies have improved our understanding of the molecular basis for the development and stress resistance of an important banana variety.

## 1. Introduction

WRKY transcription factors (TFs) are one of the largest families in plants and form integral parts of signaling webs that modulate many plant processes [1]. There are one or two conserved domains with a length of 60 amino acid residues in the amino acid sequence of WRKY transcription factors, which includes an N-terminal DNA binding motif and a C-terminal zinc finger motif. Plant WRKY transcription factors can be divided into three categories: the first family contains two WRKY domains and two zinc finger structures with C_2_H_2_; the second family contains only one WRKY domain and the zinc finger structure is C_2_H_2_; and the third family contains one WRKY domain and the zinc finger structure is C_2_HC [2]. The amino acid sequence of the zinc finger motif is CX_4–5_CX_22–23_HXH (C is cysteine, X is any amino acid, X_4–5_ is any 4–5 amino acid, X_22–23_ is any 22–23 amino acid, and H is histidine) or CX_7_CX_23_HXC (X_7_ is any 7 amino acid and X_23_ is any 23 amino acid).

In 1994, the first WRKY gene was identified from sweet potato (*Ipomoea batatas* L. Lam), and its coding protein was named the SPF1 protein [3]. With the completion of plant genome sequencing, more and more WRKY gene families have been identified at the whole genome level, such as in apple [4], lily [5], *Ricinus communis* [6], carrot [7], and cotton [8]. A large number of studies have shown that WRKY TFs play a key role in plant defense, including against bacteria [9] and fungi [10]. WRKY accumulation factors are also involved in various abiotic stressors. The expression of some WRKY transcription factors is strongly induced under abiotic stress, such as salinity, drought, low temperature, and hormone stress [11]. WRKY transcription factors play an important role in plant hormone mediated signal transduction [12,13,14,15]. Salicylic acid is one of the five hormones, and previous studies found that it is involved in the process of banana resistance to Foc TR4 [16]. In other plants, WRKY TF has been shown to regulate disease resistance by regulating salicylic acid key enzyme genes or genes in the salicylic acid signal pathway. For example, in rice, *OsWRKY6* directly activates *OsICS1* and regulates the defense response of rice to bacterial blight [17]. In *Arabidopsis* protoplasts, WRKY28 can bind to *ICS1* promoter and activates *ICS1* expression [18]. AtNAC019, AtNAC055 and AtNAC072 can repress the expression of *AtICS1* through direct interaction with its promoter [19]. AtTCP proteins activate *AtICS1* expression through direct interaction with its promoter during pathogen infection [20].

Banana (*Musa*. spp.) is a fruit product with remarkable economic benefits in the market at home and abroad, and production contributes to many people’s income [21]. The Brazilian banana (*M*. *acuminata*) is a triploid (AAA) banana cultivar. However, in the process of planting, bananas are often blocked by abiotic and biotic stress, such as drought [22], low temperature [23], salt [24], and several devastating diseases [25]. Banana Fusarium Wilt is one of the most important diseases of banana and one of the most serious plant diseases in the world and is caused by *Fusarium oxysporum* f. sp. *cubense* (Foc). Foc tropical race 4 (Foc TR4) is a disease that is destructive to the development of the banana industry [26]. *M*. *acuminata* (A genome) [27] and *M*. *balbisiana* (B genome) [28] are the main parents of edible banana and have been sequenced. Thus far, although there are two reports focused on the WRKY gene family, these data are based on version 1 of the *M*. *acuminata* genome [29,30]. In 2016, the *M*. *acuminata* genome reference was improved by a combination of methods and datasets. Compared with version 1 of the *M*. *acuminata* genome, the assembly of the genome sequence and gene annotation has changed greatly in version 2 [31]. Until now, no systematic information has been available regarding the WRKY family in version 2 of the *M*. *cuminate* genome. The WRKY family has been associated with plant senescence, organ development, and stress tolerance in many plants. In this study, we systematically analyzed the WRKY gene family based on version 2 of the *M*. *acuminata* genome to understand its potential relevance to fruit ripening and stress tolerance and demonstrated that MaWRKY21 can directly bind to the W-box of the *MaICS* promoter to decrease *MaICS* transcription and, in turn, enzyme activity. This new comprehensive study can improve our understanding of *MaWRKYs*-related fruit development, ripening processes, and stress responses and can establish a base for future studies of genetic improvement in banana.

## 2. Results

### 2.1. Identification Analysis of MaWRKYs

A total of 164 sequences were identified as putative members of the *MaWRKY* gene family. The 164 predicted MaWRKY proteins ranged from 114 (MaWRKY153) to 828 (MaWRKY74) amino acid residues with a relative molecular mass varying from 13.56 (MaWRKY153) to 90.05 kDa (MaWRKY74), and the theoretical isoelectric points (PIs) were predicted to range from 4.80 (MaWRKY142) to 10.16 (MaWRKY17) (Table S1).

### 2.2. Phylogenetic Analysis of MaWRKYs

**Figure 1:**
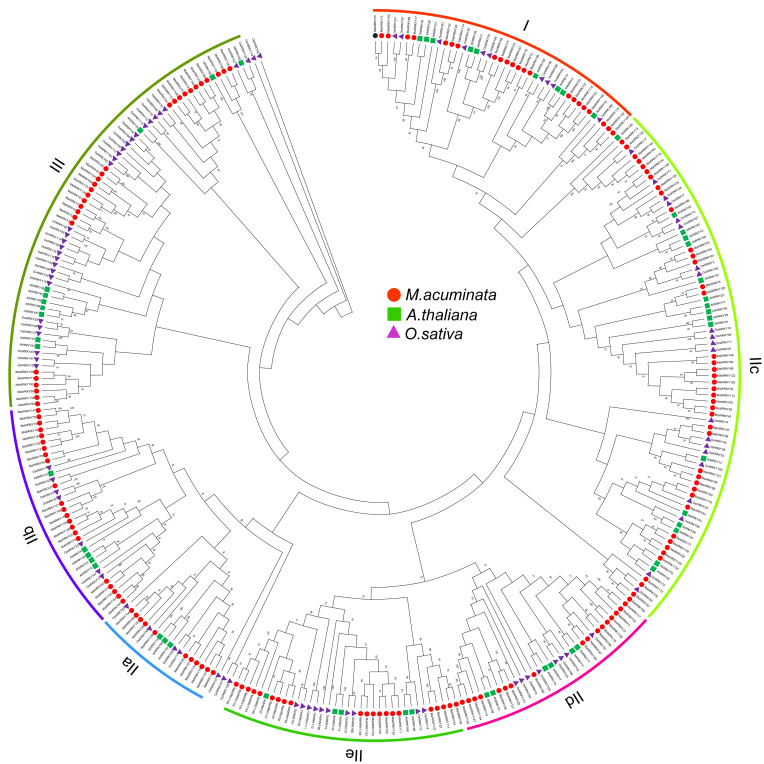
Phylogenetic tree of WRKY TFs constructed based on WRKY domains from *Arabidopsis* (in green and prefised by At) and rice (in purple and prefised by Os), and *M. acuminata* (in red and prefised by Ma).

To analyze the evolutionary relationships among MaWRKYs, 164 MaWRKY proteins were aligned with AtWRKY proteins from *Arabidopsis* and OsWRKY proteins from rice, and an unrooted phylogenetic tree was constructed by MEGA7. WRKY conservative domains can be divided into three categories: I, II, and III, according to Eulgem [2]. Among them, group I contains two WRKY domains and groups II and III contain one WRKY domain; groups I and II have a C_2_H_2_ type zinc finger domain and group III has a C_2_HC domain. There are 23 members in group I, 113 members in group II, and 28 members in group III. According to the classification in *Arabidopsis*, group II was further classified into subclasses IIa (11), IIb (23), IIc (33), IId (9), and IIe (29) (Figure 1).

### 2.3. Chromosome Location and Repeat Sequence of MaWRKYs

**Figure 2:**
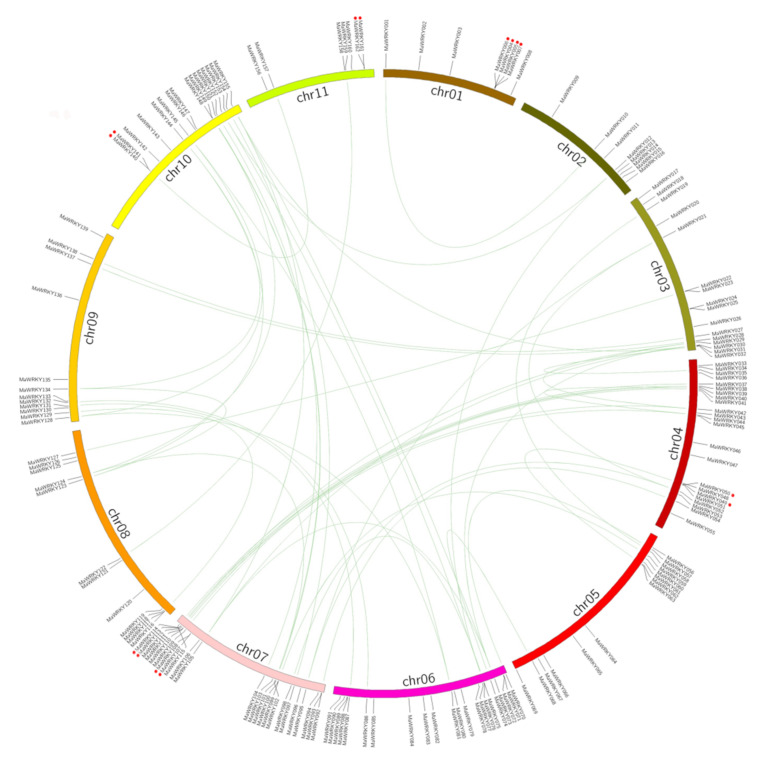
Distribution and synteny analysis of MaWRKYs on the 11 chromosomes. The vertical black line indicates the position of MaWRKY. The segmental duplicate MaWRKYs are connected with green lines. Tandem duplicates were marked with red dots.

Overall, 162 MaWRKYs were found to be distributed on 11 chromosomes using genome chromosomal location analyses (Figure 2), and the number of MaWRKYs was different across chromosomes. Among them, there were 24 on chromosome 7, 23 on chromosome 4, and 22 on chromosome 6. There were at least 7 on chromosome 11, 8 on chromosomes 1 and 2, 12 on chromosomes 8 and 9, 16 on chromosomes 3 and 10, and 14 on chromosome 5. Gene duplication is an important source of new gene generation and function evolution. Gene duplication can be divided into two categories: tandem duplication refers to duplication events in which duplication genes are located on the same chromosome, and there are no other genes between them. Duplication events with duplication genes located on different chromosomes or at a relatively distant position on the same chromosome are called segmental duplications. In the *M.* *acuminata* genome, 12 conserved collinear blocks were found [27]. We found that 71 genes were located in 12 collinear blocks and there were 31 group sets of segmental duplication and 7 pairs of gene tandem duplication (Figure 2 and Table S2).

### 2.4. Sequence Analysis of the Conserved Domain MaWRKY Proteins

**Figure 3:**
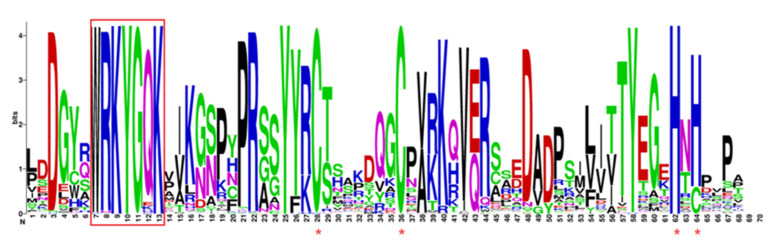
WRKY gene family conserved domain, WRKYGQK conserved domain in the red box, red asterisk refers to zinc finger structure.

All 164 MaWRKY protein sequences were found to contain WRKY domain using CD-Search in NCBI and ClustalW align. The longest conserved domain was composed of 828 amino acids. The domain of MaWRKYs was found to be highly conserved with occasional variants (Figure 3). Among them, the C-terminal WRKY heptapeptide domain and zinc finger structure of class I were WRKYGQK and CX_4_CX_23_HXH, and the N-terminal WRKY heptapeptide domain and zinc finger structure were WRKYGQK and CX_4_CX_22_HXH, which was more similar to the C-segment zinc finger. In class II subclasses a, b, c, d, and e, the WRKY heptapeptide domain and zinc finger structure were WRKYGQK and CX_5_CX_23_HXH (except for MaWRKY117, in which the WRKY heptapeptide domain was WRKYGEK). The structure of class IIc heptapeptide domain and zinc finger was WRKYGQK and CX_4_CX_23_HXH (except for MaWRKY25, MaWRKY98, and MaWRKY109, in which the WRKY heptapeptide domain was WRKYGKK; in MaWRKY40, the WRKY heptapeptide domain was WRKYGRK; in MaWRKY53 and MaWRKY94, the WRKY heptapeptide domain was WNKYGRK). In class III, the heptapeptide domain and zinc finger structure were WRKYGQK and CX_7_CX_23_HXC (except for MaWRKY41, in which the WRKY heptapeptide domain was WRKYGHK; in MaWRKY4, MaWRKY5, MaWRKY6, and MaWRKY7, the WRKY heptapeptide domain was WRKYGEK).

### 2.5. Analysis of the Expression Profile of MaWRKYs in Different Development and Postharvest Ripening Stages of Banana Fruit

**Figure 4:**
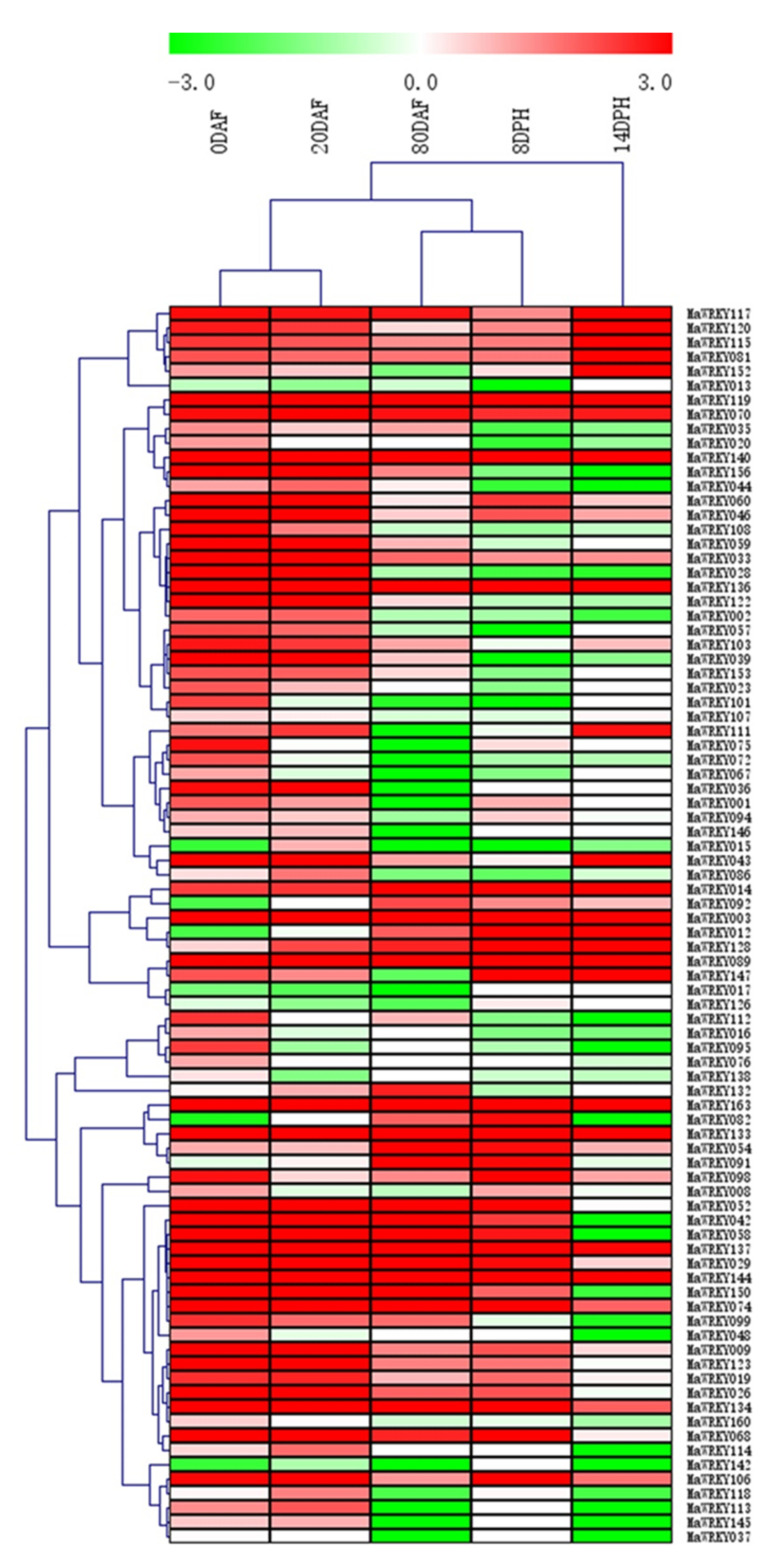
Heatmap of differentially expressed *MaWRKYs* in five tissues during fruit development and ripening.

To evaluate the expression characteristics of *MaWRKYs*, RNA-Seq data were obtained from the fruit development and ripening stages (Table S3). Among the 164 *MaWRKYs*, 42 and 38 showed high expression levels at 0 days after flowering (DAF) and 20 DAF (RPKM > 5), accounting for 25.6 and 23.2% of the total genes, respectively, and 24, 27, and 23 *MaWRKYs* were highly expressed at 80 DAF, and 8 and 14 days postharvest (DPH) (RPKM > 5), accounting for 14.6, 16.5, and 14.0% of the total genes, respectively (Figure 4 and Table S4). Among them, *MaWRKY3*, *MaWRKY70*, *MaWRKY119*, *MaWRKY133*, *MaWRKY137*, *MaWRKY140*, *MaWRKY144*, and *MaWRKY163* were highly constitutively expressed at 0, 20, and 80 DAF and 8 and 14 DPH (Figure 4 and Table S4), indicating that these genes play regulatory roles during fruit development and postharvest ripening stages.

### 2.6. Expression Profile Analysis of MaWRKYs under Abiotic Stress and Foc TR4 Treatment

**Figure 5:**
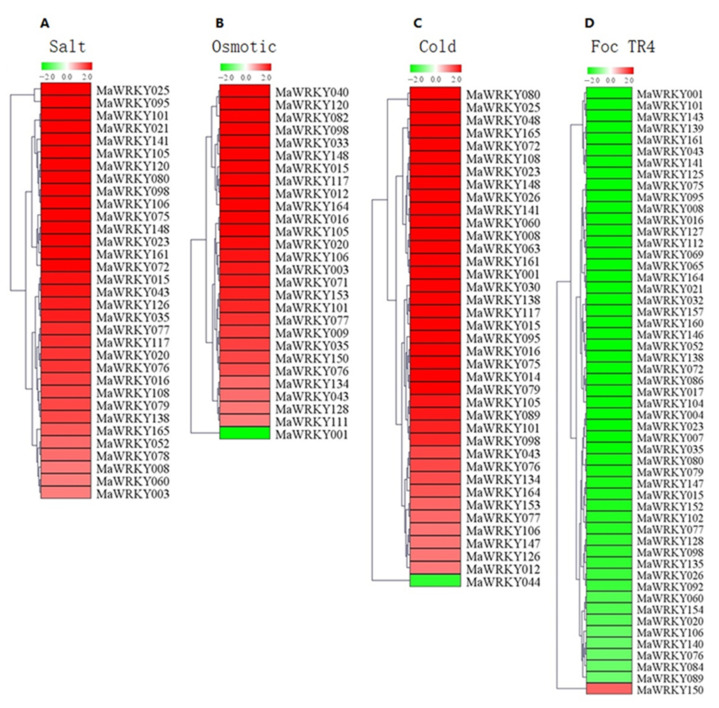
Expression patterns of *MaWRKYs* in response to abiotic and biotic stress. (**A**) salt; (**B**) osmotic; (**C**) cold; (**D**) Foc TR4.

**Figure 6:**
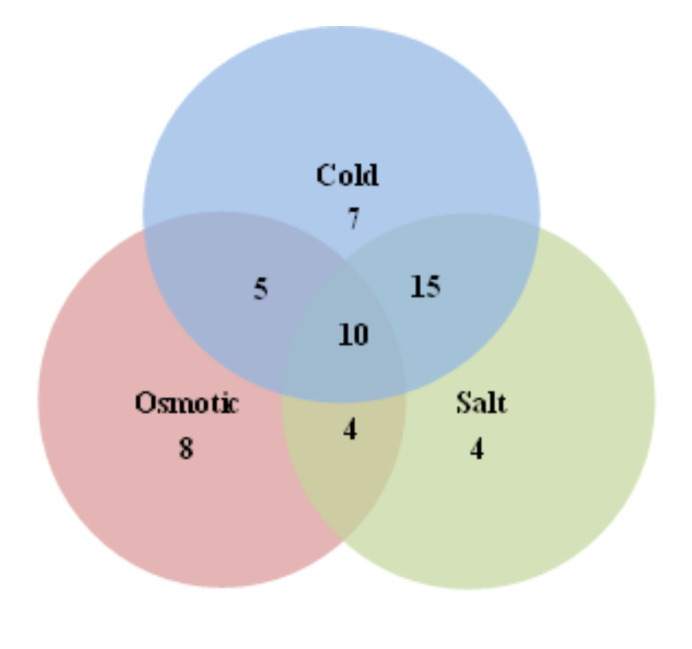
The number of genes involved in two or three abiotic stresses at the same time.

RNA-Seq data were used to analyze the expression profiles of *MaWRKYs*; these data were obtained from banana plantlets in response to osmotic, salt, cold, and Foc TR4 treatments (Table S3). We deleted genes with RPKM values less than 5 to present the differentially expressed genes intuitively and accurately. The cold treatment (4 °C) resulted in 39 *MaWRKYs* being differentially expressed (log2 Ratio Cold/Control > 1), of which 38 were upregulated and one was downregulated (Figure 5 and Table S5). Drought treatment (200 mM mannitol) resulted in 28 *MaWRKYs* being differentially expressed (log2 Ratio Osmotic/Control > 1), of which 27 were upregulated and one was downregulated (Figure 5 and Table S6). Salt treatment (300 mM NaCl) resulted in 33 *MaWRKYs* being differentially expressed (log2 Ratio Salt/Control > 1), and all were upregulated (Figure 5 and Table S7). Under abiotic stress, 10 genes (*MaWRKY15*, *MaWRKY16*, *MaWRKY43*, *MaWRKY76*, *MaWRKY98*, *MaWRKY100*, *MaWRKY105*, *MaWRKY106*, *MaWRKY117*, and *MaWRKY152*) responded to 3 abiotic stressors at the same time, 15 genes responded to cold and salt stress, 5 genes responded to cold and drought stresses, and 4 genes responded to osmotic and salt stress (Figure 6). These results indicate that *WRKY* genes are mainly involved positively in banana’s response to abiotic stress. 

Foc TR4 treatment resulted in the differential expression of 53 *MaWRKYs* (log2 Ratio 2 DPI/0 DPI > 1), 1 that was upregulated and 52 that were downregulated (Figure 5 and Table S8). The results show that these 53 differentially expressed *MaWRKYs* may play a role in the interaction between banana and Foc TR4.

### 2.7. Expression Patterns of MaWRKYs during the Interaction between Banana and Foc TR4

**Figure 7:**
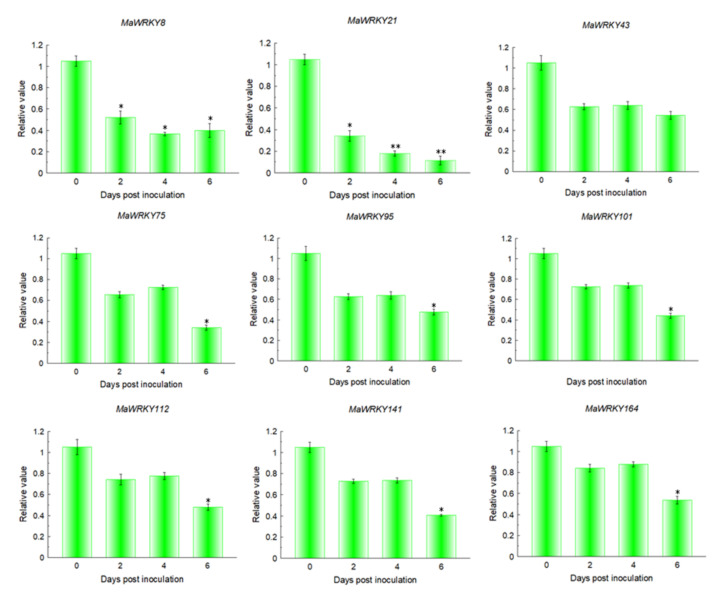
Expression patterns of nine *MaWRKYs* under Foc TR4 treatment by qRT-PCR. The data represent the mean ± standard deviation (SD) of three replicates. * and ** significantly different from the control at *p* < 0.05 and 0.01, respectively.

Nine differentially expressed *MaWRKYs* (*MaWRKY8*, *MaWRKY21*, *MaWRKY43*, *MaWRKY75*, *MaWRKY95*, *MaWRKY101*, *MaWRKY112*, *MaWRKY141*, and *MaWRKY164*) were selected to analyze their expression patterns in Brazilian banana to Foc TR4 infection by quantitative real-time (qRT)-PCR. The roots of banana plantlets without Foc TR4 treatment were used as a control. RNA was extracted from the roots of Brazilian banana at 2, 4, and 6 DPI (days postinfection) and in the control. In the Brazilian banana, the expression levels of nine *MaWRKYs* were decreased at 2, 4, and 6 DPI. We found that nine *MaWRKYs* displayed the same characteristics between the RNA-Seq data and qRT-PCR data at 0 and 2 DPI (Figure 7). These results indicate that these genes are involved in the interaction between plantain and Foc TR4.

### 2.8. Interaction Network of MaWRKY21

**Figure 8:**
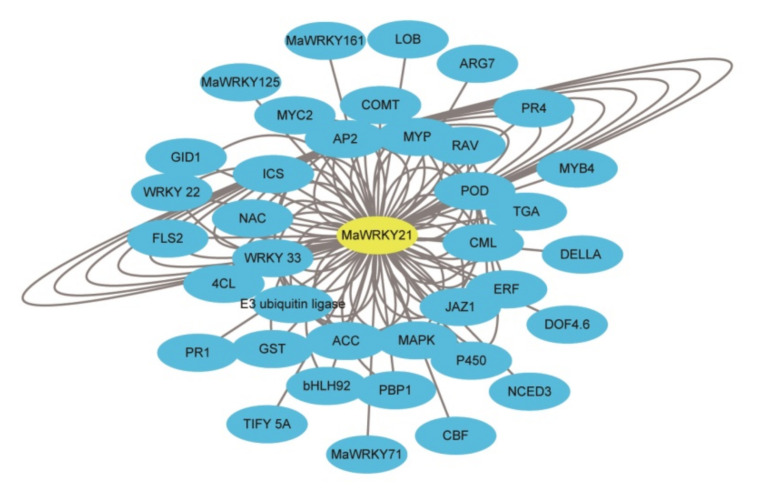
The interactive networks of *MaWRKY21* using Cytoscape.

Owing to the lowest expression level of *MaWRKY21* in response to Foc TR4, we selected it to construct a co-expression network to further explore the regulatory network using Cytoscape [32,33]. In total, 106 interactive proteins for MaWRKY21 were detected (Figure 8 and Table S10). The 106 key proteins that interacted with MaWRKY21 were divided into 36 classes, including the most abundant TF family AP2/EREBP (22), followed by POD (peroxidase) (8), MYB (Myb-related protein) (5), ACC (1-aminocyclopropane-1-carboxylate synthase) (5), WRKY33 (5), E3 ubiquitin-protein ligase (5), WRKY22 (4), RAV-like factor (4), MAPK (mitogen-activated protein kinase) (3), JAZ1 (jasmonate ZIM domain-containing protein) (3), CML (calcium-binding protein CML) (3), COMT (caffeic acid O-methyltransferase) (3), ERF (ethylene-responsive transcription factor) (3), NAC (3), TGA (2), PBR1 (calcium-binding protein PBP1) (2), MYC2 (2), 4CL (4-coumarate-CoA ligase) (2), GST (glutathione S-transferase) (2), P450 (2), bHLH (2) with single-member for ICS (isochorismate synthase), DOF4.6 (dof zinc finger protein DOF4.6-like), DELLA protein, NCED3, ARG7 (indole-3-acetic acid-induced protein ARG7-like), CBF, FLS2 (LRR receptor-like serine/threonine-protein kinase FLS2), GID1 (gibberellin receptor), MaWRKY125, MaWRKY71, MaWRKY161, LOB, TIFY 5A, PR4 (pathogenesis-related protein 4), and PR1 (pathogenesis-related protein 1). The results indicate that these proteins, together with MaWRKY21, may play a role in the response of banana to Foc TR4 infection.

### 2.9. Binding of MaWRKY21 to the MaICS Promoter

**Figure 9:**
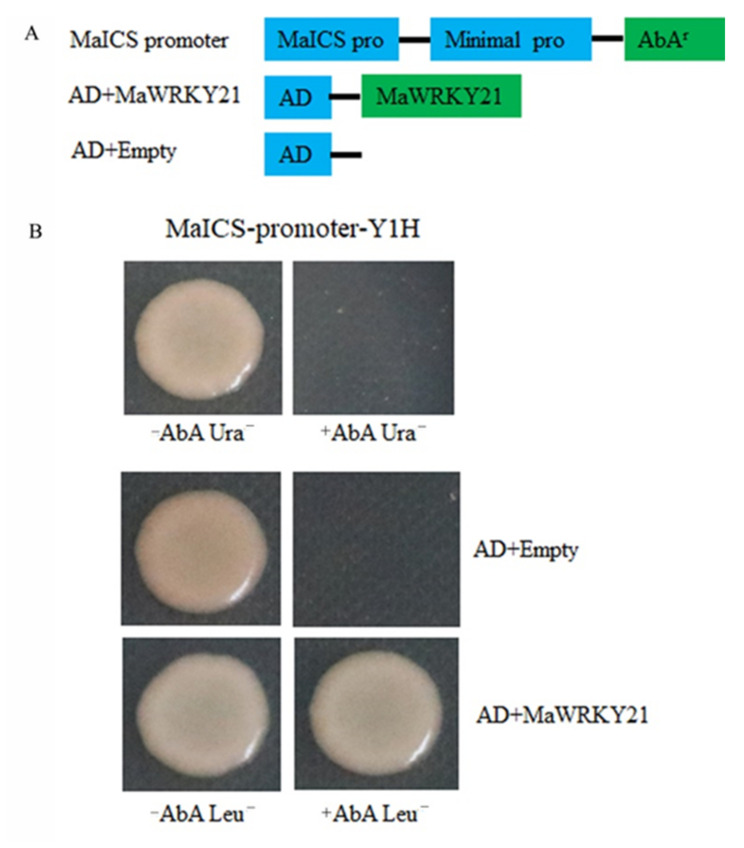
The interaction between *MaWRKY21* and *MaICS* promoter by Y1H assay. (**A**) Schematic diagram vectors for Y1H assay. (**B**) Y1H assay.

Previous studies found that salicylic acid is involved in the process of banana resistance to Foc TR4 [16]. Isochorismate synthase is one of the key enzymes in salicylic acid biosynthesis and the rate limiting enzyme in salicylic acid biosynthesis [34]. Therefore, *MaICS* was selected for yeast one hybrid verification, which was associated in the co-expression network. The 1692 bp upstream sequence of *MaICS* was isolated from banana genomic DNA according to the banana *M*. *acuminata* genome, and the 1692 bp main regulatory elements of the *MaICS* promoter were predicted online by plantcare websit. We found that there was a conserved W-box element bounding to WRKY TF in the *MaICS* promoter sequence. The *MaICS* promoter was cloned into the pAbAi vector, generating pAbAi-*MaICSp*. *MaWRKY21* was cloned into the pGADT7-AD vector, generating pGADT7-*MaWRKY21* (Figure 9A). The yeast one hytrid (Y1H) Gold yeast co-transformed with pAbAi-*MaICS*p and pGADT7-*MaWRKY21* could grow normally on the SD/-Leu medium containing 300 ng/L AbA, whereas the pAbAi-*MaICS*p and pGADT7-AD could not grow (Figure 9B), which proved that *MaWRKY21* can directly bind to the sequence of the *MaICS* promoter.

### 2.10. Transcriptional Regulation of MaWRKY21 on MaICS

**Figure 10:**
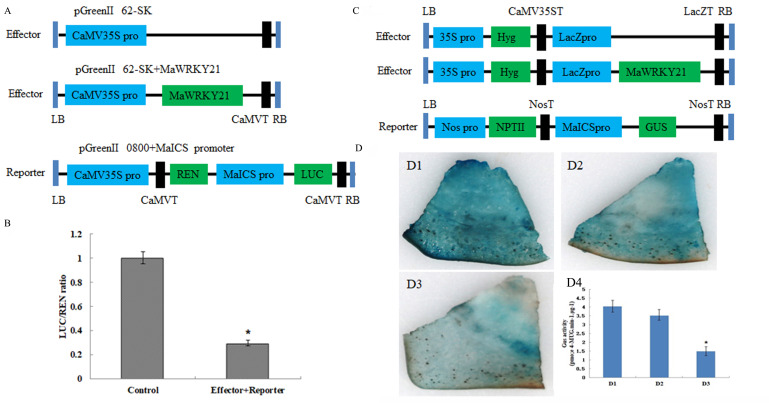
The interaction between MaWRKY21 and *MaICS* promoter by LUC/REN and GUS assay. (**A**) Schematic diagram vectors for LUC/REN assay. (**B**) Relative LUC/REN ratio. (**C**) Schematic diagram vectors for GUS assay. (**D**) GUS staining in banana fruits and GUS activity. (**D1**) Positive control; (**D2**) *MaICS* promoter; (**D3**) MaWRKY21 + reporter; (**D4**) GUS activity. * significantly different from the control at *p* < 0.05.

The date above showed that MaWRKY21 can bind to the *MaICS* promoter, but it remains to be determined whether it is an activator or a repressor. Therefore, we used the dual luciferase (LUC) assay to investigate how MaWRKY21 interacts with the *MaICS* promoter. The reporter vector (MaICSp:LUC) and effector (MaWRKY21) were co-infected in tobacco leaves epidermal cells by *Agrobacterium*-mediated transformation, and the relative LUC activity was determined. The results showed that, compared with the control (empty load of reporter gene and effector gene), the fluorescence of LUC decreased significantly, indicating that MaWRKY21 may function as a transcriptional repressor. (Figure 10A, B). We further used the GUS reporting system to study how MaWRKY21 interacts with the *MaICS* promoter. The PBI121 empty vector and PBI121-*MaICS* vector were transformed into banana fruit slices, MaWRKY21 and the PBI121-*MaICS* were co-transformed in banana fruit slices. β-glucuronidase (GUS) staining and activity assays showed that GUS activities were lower in the banana fruits slices with MaWRKY21 + PBI121-*MaICS* than in slices infected with PBI121 and PBI121-*MaICS*. The results showed that *MaWRKY21* may negatively regulate the expression of *MaICS* (Figure 10C, D). These results of the GUS staining system and double luciferase activity showed that MaWRKY21 could inhibit the expression of *MaICS.*

## 3. Discussion

WRKY TFs play pivotal regulator roles in plant growth, development, and response to a variety of abiotic and biotic stressors [35]. The WRKY gene family in plants is large. There are 72 *WRKYs* in *Arabidopsis*, 96 in rice, 55 in cucumber, and 197 in soybean. In previous research reports, 153 WRKY genes were identified in banana with reference to the first version of the *M*. *acuminata* genome and 6 genes were not located on a chromosome [29]. In total, 147 WRKY genes were identified in banana with reference to the first version of the *M*. *acuminata* genome and 6 genes were not located on a chromosome [30]. Based on the second version of the *M.* *acuminata* genome, we identified 164 WRKY genes and 2 genes that were not located on a chromosome. In our study, there were 11 and 17 more genes than in the original report. Three genes assembled on the first version of the *M*. *acuminata* genome do not appear in the second version (*GSMUA_Achr4G14270_001*, *GSMUA_Achr6G03810_001*, and *GSMUA_Achr10G02060_001*). In the first version of the *M*. *acuminata* genome, four genes were not located on any chromosome, while in the second version of the *M*. *acuminata* genome, they were located on the chromosome (for example: *GSMUA_AchrUn_randomG17570_001* is located on the second chromosome reference to the second version of the *M*. *acuminata* genome and named *Ma02_g00790*). The first version of the *M*. *acuminata* genome was compared with the second version of the *M*. *acuminata* genome, the chromosomal localization of seven genes was changed (for example: *GSMUA_Achr1G04770_001* is located on the fourth chromosome reference to the second version of the *M*. *acuminata* genome and named *Ma04_g24540*) (Table S11). The results showed that with the development of bioinformatics, the second version of the banana *M*. *acuminata* genome was assembled more accurately. The quality of genome assembly is an important factor affecting the number of genes.

Resistant (Karthobiumthum (ABB) (NRCB-0050)) and susceptible (Nendran (AAB) (NRCB-0615)) varieties were inoculated with a nematode; the expression profile of the *MaWRKY* family was analyzed using transcriptome data, and six genes were verified by fluorescence quantitative PCR [29]. Goel [30] analyzed the expression profile of the *MaWRKY* family with reference to the published transcriptome data (fruit transcriptome datasets of *M*. *acuminata*; dwarf Cavendish; root transcriptome datasets of Brazilian), and the expression of five genes was analyzed in fruit development, maturity, and in different organs of dessert (*M*. *acuminata*) and cooking (*M*. *paradisiaca)* varieties using fluorescence quantitative PCR. In our study, we analyzed the transcriptome of the fruit development stage, and seedlings under cold stress, salt stress, drought stress, and Foc TR4 stress in Brazilian banana. Additionally, the expression of nine *MaWRKYs* was analyzed after Brazilian banana was stressed by Foc TR4. Our results enrich the understanding of the WRKY family in bananas.

WRKY is a class of plant-specific TFs which typically contains 1–2 WRKY domains composed of about 60 highly conserved amino acids. This domain contains a highly conserved core sequence of WRKYGQK at the N-terminal and a variable zinc finger structure at the C-terminal [29]. It regulates gene expression by specifically binding to the W-box in the promoter region of the target gene. We found that 164 MaWRKY proteins contained at least one conservative WRKY domain. In *Caragana intermedia*, the seven amino acids of WRKYGQK in the WRKY TF were mutated [36]. In our study, we also found that MaWRKY53 and MaWRKY94 had a mutated amino acid sequence, and the WRKY motif changed to WNKY. In the GQK motif, 12 genes changed to GEK (MaWRKY4, MaWRKY5, MaWRKY6, MaWRKY7, and MaWRKY117), GKK (MaWRKY25, MaWRKY98, MaWRKY109, and MaWRKY126), GHK (MaWRKY41 and MaWRKY113), and GRK (MaWRKY40). In soybean, GmWRKY6 and GmWRKY21 contained the WRKYGKK motif and could not normally bind to the W-box [37]. In tobacco, NtWRKY12 has the WRKYGQK motif, and can bind specifically to the sequence TTTTCCAC instead of the normal W-box [38]. We presumed that the variation in the WRKYGQK motif could lead to a significant decrease or even complete loss of binding ability to the W-box, or that it could bind to other new motifs and generate new functions.

Gene duplication events play an important role in the amplification and evolution of plant gene families [39]. The *M*. *acuminata* genome of banana has experienced three genome-wide duplication (WGD) events (α-, β-, and γ-WGD), which led to duplicated segments included in the *Musa* ancestral blocks, covering 222 Mb and containing 26,829 genes [27]. There are many homologous genes of banana *WRKYs* in each sub-branch, which indicates that the banana genome retained more homologous genes in the process of diploidization after WGD. In *Arabidopsis* [40], carrot [7], peanut [41], and tomato [42], WRKY gene expansion occurs mainly by tandem duplication. In our study, 30.5% (50/164) of *MaWRKYs* were located in collinear regions (Figure 2 and Table S2), which indicated that the expansion of banana *WRKYs* was caused by segmental duplication.

WRKY TFs are involved in fruit development and ripening. In watermelon, 33% of *CLWRKY* were expressed in fruit, and some of them were positively or negatively involved in regulatory pathways of plant hormones, thus playing important roles in plant growth, development, and defense against environmental stress [43]. In banana, analysis of the mature and immature fruits by transcriptome sequencing showed that some *MaWRKYs* were highly expressed in mature fruits, while some *MaWRKYs* were lowly expressed during fruit ripening, indicating that *MaWRKYs* had different roles in fruit development and ripening [44]. In our results, 50% of the *MaWRKYs* were highly expressed in fruit ripening (Figure 4), indicating that *MaWRKYs* played important roles in fruit ripening.

*WRKYs* are involved in the plant response to abiotic and biotic stress [45]. Overexpression of *CsWRKY46* from cucumber in *Arabidopsis* enhanced the cold resistance of transgenic lines and increased the expression of cold related genes *RD29A* and *COR47*, showing that *CsWRKY46* played a role in cold stress resistance in cucumber [46]. In tobacco, overexpression of cotton *GhWRKY25* reduced tolerance to drought and increased salt tolerance [47]. Overexpression of *DgWRKY1* or *DgWRKY3* from *Dendranthema grandiflorum* in tobacco enhanced the salt tolerance of transgenic lines [48]. *MaWRKY31*, *MaWRKY33*, *MaWRKY60*, and *MaWRKY71* can directly bind to the W-box elements of the promoters of *MaNCED1* and *MaNCED2* genes and activate their expression, which can enhance the cold tolerance of banana fruit by increasing the content of endogenous ABA (abscisic acid) [49]. In our studies, we found that more than 17.1% of *MaWRKYs* were differentially expressed under cold, drought, and salt stress, and 10 genes (*MaWRKY15*, *MaWRKY16*, *MaWRKY43*, *MaWRKY76*, *MaWRKY98*, *MaWRKY100*, *MaWRKY105*, *MaWRKY106*, *MaWRKY117*, and *MaWRKY152*) responded to three abiotic stressors at the same time (Figure 6). These results indicate that *MaWRKYs* play important roles in banana’s response to drought, high salinity, and low temperature.

The WRKY family also plays an important role in plant disease resistance. In banana, MaNAC5 interacted with MaWRKYs to enhance the expression of pathogenesis-related genes against *Colletotrichum musae* [50]. Overexpression of *GhWRKY39-1* enhanced the resistance of transgenic tobacco to the bacterial pathogen *Ralstonia solanacearum* and fungal pathogen *Rhizoctonia solani* [51]. In *Arabidopsis,* overexpression of wild grape WRKY transcription factor *VqWRKY52* enhanced the resistance of transgenic *Arabidopsis* to the nutritional pathogen powdery mildew [52]. We found that 53 *MaWRKYs* were differentially expressed after inoculation with Foc TR4, and 52 genes were downregulated and only 1 gene was upregulated (Figure 5 and Table S8). qRT-PCR results suggested that *MaWRKY8*, *MaWRKY21*, *MaWRKY43*, *MaWRKY75*, *MaWRKY95*, *MaWRKY101*, *MaWRKY112*, *MaWRKY141*, and *MaWRKY164* were decreased at 2, 4, and 6 DPI. The results showed that these genes were involved in the interaction between plantain and Foc TR4.

In this study, the co-expression network of MaWRKY21 is associated with *MaICS*, which is the key gene of salicylate synthase, implying that MaWRKY21 might be responsible for the expression of *MaICS* in the biosynthesis of salicylic acid. Before our laboratory analyzed the expression of *MaICS* in resistant and susceptible varieties, the results showed that salicylic acid played a role in the interaction between banana and Foc TR4 [16]. However, there is no study about the regulation mechanism of *MaICS* in banana, so we selected the gene to study whether MaWRKY21 has a regulatory effect on it. The results showed that MaWRKY21 can bind with the promoter of *MaICS* and negatively regulate the expression of *MaICS* (Figure 9 and Figure 10). In other plants, the regulatory mechanism of *ICS* has been reported. In *Arabidopsis* protoplasts, WRKY28 can bind to the *ICS1* promoter and activate *ICS1* expression [18]. AtNAC019, AtNAC055, and AtNAC072 can repress the expression of *AtICS1* through direct interaction with its promoter [19]. AtTCP proteins activate *AtICS1* expression through direct interaction with its promoter during pathogen infection [20]. In rice, OsWRKY6 directly activates *OsICS1* and regulates the defense response of rice to bacterial blight [17]. These results showed that the regulatory mechanism of *ICS* is very complex, there may be many factors regulating it in the same plant. In this study, we demonstrated that MaWRKY21 can regulate the expression of *MaICS*. As there is a complex coordinated regulatory mechanism underlying *ICS* expression, there may be other proteins regulating the expression of *MaICS* in banana.

## 4. Materials and Methods

### 4.1. Identification of WRKY Family Genes in Banana

Whole protein sequences of the *M*. *acuminata* genome (DH-Phang) were obtained from the banana genome database (http://banana-genome.cirad.fr/). Subsequently, the iTAK program (http://itak.feilab.net/cgi-bin/itak/index.cgi, accessed on 9 October 2020) was used to identify TFs according to the consensus rules, which are mainly summarized within PlnTFDB and PlantTFDB [53,54], and obtained all candidate MaWRKY protein sequences. Finally, all candidate MaWRKY protein sequences were further evaluated using BLASTp and CDD (http://www.ncbi.nlm.nih.gov/cdd/, accessed on 2 November 2020) databases in NCBI. Fifty-three WRKY protein sequences of *Arabidopsis* and 95 WRKY protein sequences of rice were obtained from TAIR (http://www.arabidopsis.org/, accessed on 5 November 2020) and RGAP (http://rice.plantbiology.msu.edu/, accessed on 5 November 2020) databases.

The WRKY protein sequences of banana, *Arabidopsis*, and rice were aligned using Clustal X2.0, the phylogenetic tree was constructed using MEGA 7.0 with 1000 bootstraps. Repeat sequences of WRKY were analyzed by Circos (http://circos.ca/, accessed on 5 November 2020). The molecular weight and isoelectric points of MaWRKYs were predicted from the ExPASy database (http://expasy.org/, accessed on 8 November 2020) (Table S1). The sequence logo for the WRKY domain was constructed by the WebLogo server (http://weblogo.berkeley.edu/logo.cgi, accessed on 2 February 2022).

### 4.2. Chromosome Localization and Gene Duplications

To resolve the physical localizations of *MaWRKYs*, the beginning and terminative positions of all *MaWRKYs* on each chromosome were obtained from the banana *M*. *acuminata* genome database. The image of the locations of the banana WRKY genes was drawn using MapInspect software (http://mapinspect.software.informer.com/, accessed on 10 December 2020). Tandem and segmental duplications were determined according to the plant genome duplication database [55]. Syntenic blocks were discovered using MCSCAN [28]. The image of locations and synteny of the *MaWRKYs* was drawn using Circos (0.63) software.

### 4.3. Plant Materials and Treatments

The banana fruits were obtained from the Banana Plantation of the Institute of Tropical Bioscience and Biotechnology (Chengmai, Hainan, 20 N, 110 E). To analyze expression at the developmental and mature stages of banana fruit, developing banana fruits at 0, 20, and 80 DAF were collected from BX banana, representing fruit developmental stages of budding, cutting flowers, and harvest stages, respectively. To analyze the expression of *MaWRKYs* during the postharvest ripening processes, fruits at 8 (more green than yellow) and 14 (full yellow) DPH (days postharvest) in BX were collected and used for postharvest analyses.

One-month-old banana (*Musa acuminata* L. AAA group, cv. Brazilian) plantlets were grown in Hoagland’s solution [56] under greenhouse conditions with 70% relative humidity and at 28 °C, 16 h light/8 h dark cycle. For salt treatment, 15 banana plantlets at the five-leaf stage were irrigated with 300 mM NaCl for 7 days. For osmotic treatment, 15 banana plantlets at the five-leaf stage were irrigated with 200 mM mannitol for 7 days. For cold treatment, 15 banana plantlets at the five-leaf stage were incubated at 4 °C for 22 h. Leaves without major veins were harvested for analysis. For Foc TR4 treatment, 15 banana plantlets at the five-leaf stage roots were incubated in a Foc TR4 spore suspension of 1.5 × 10^6^ condia/mL, the entire root system was harvested at 0, 2, 4, and 6 DPI. All samples were immediately frozen in liquid nitrogen and stored at −80 °C until RNA extraction transcriptome sequencing and gene expression analysis.

### 4.4. Transcriptome Analysis

Total RNA was extracted using RNA extraction kit (TIANGEN, Beijing, China) and converted into cDNA using a cDNA Synthesis Kit (Fermentas, Beijing, China). The TruSeq RNA library preparation kit v2 was used to construct the cDNA library, then it was sequenced on the IIIumina HiSeq 2000 platform (San Diego, CA, USA) using the IIIumina RNA-Seq protocol. Three biological replicates were used for each sample. Reads per kilobases of exon model per million reads (RPKM) were considered as gene expression levels [57]. Differentially expressed genes were evaluated with DEGseq [58]. A heat map was created with MeV 4.9 and Java Treeview software according to the manufacturer’s protocol. All RNA-Seq data were deposited in the CNSA (https://db.cngb.org/cnsa/, accessed on 6 September 2019) of CNGBdb [26].

### 4.5. qRT-PCR

According to the manufacturer’s protocol (Fermentas, Beijing, China), 1 μg RNA was used for cDNA synthesis using the first-strand reverse transcription kit with oligo-(dT) primers. The first strand cDNA was diluted (1:5) with water and used as a template for qRT-PCR. Nine *MaWRKYs* were detected by qRT-PCR analysis. Specific gene primers (Table S9) were designed using PRIMER5 software. *MaActin* (Genebank accession numbers: EF672732) was used as an internal control to normalize the relative expression of the target genes. The relative expression levels of *MaWRKYs* were assessed based on the 2^−ΔΔCt^ method [59]. Each sample contained three replicates.

### 4.6. Construction of Regulatory Networks

Based on the banana genome database and transcriptome analysis, the co-expression network of MaWRKY21 was extracted and the network connections were visualized using Cytoscape software v.3.4.0 [30,31].

### 4.7. Y1H Assay

The *MaICS* promoter (1692 bp) was amplified by PCR with forward primer 5’-CCCAAGCTTACTGTCCTTTTGGGGAAG-3’ (with the H*ind* III site) and reverse primer 5’-CCGCTCGAGGGACAGCCAACAACTCCG-3’ (with the X*ho* I site) and then cloned into H*ind* III and X*ho* I restriction sites of the Y1H bait vector pAbAi (Clontech, Mountain View, CA, USA), generating pAbAi-*MaICSp*. *MaWRKY21* was amplified by PCR with forward primer 5’-CGGAATTCATGGGATCGGCTTGGTTG-3’ (with the E*coR* I site) and reverse primer 5’-CGGGATCCTTAAAGCATCATCCCGGA-3’ (with the B*amH* I site) and then cloned into E*coR* I and B*amH* I restriction sites of pGADT7, generating pGADT7-*MaWRKY21*. The Y1H experiment was conducted according to the instructions of the manufacturer using Matchmaker Gold Y1H System (Clontech, Mountain View, CA, USA). DNA–protein interactions were determined according to co-transformed growth on SD/-Leu medium supplemented with aureobasidin A. The transformed yeast cells were cultured on plates of SD/-Leu/AbA^300^ at 30 °C for 3–5 days.

### 4.8. Dual-Luciferase Reporting System Assay

The promoter of *MaICS* (1692 bp) was cloned into a pGreenII 0800-LUC vector to generate reporter vector (*MaICSp*:*LUC*). The full-length coding region of *MaWRKY21* was connected with pGreenII 62-SK to construct an effector vector. The vector was transformed into *Agrobacterium tumefaciens* strain GV3101 containing the pSoup auxiliary plasmid by the heat shock method. The reporter and effector constructs were co-transformed into tobacco leaves. After the injected tobacco plants were cultured in a light incubator for 48 h, luciferase (LUC) and Renilla luciferase (REN) were measured using the Dual-Luciferase Reporter Assay System (Promega, Madison, WI, USA). The promoter activity was assessed based on the LUC/REN ratio. Ten biological replicates were employed for each combination.

### 4.9. GUS Reporting System Assay

To verify whether MaWRKY21 could regulate the *MaICS* promoter, GUS transactivation assays were performed in banana fruit slices as described by Liu [60]. The full-length CDSs of *MaWRKY21* were cloned into PVKH, generating the effector vector, *35S*:*MaWRKY21*. The promoter of *MaICS* was cloned into the pBI121 vector, generating reporter vector *MaICSp*:*GUS*. The effector and reporter vectors were co-transformed into banana fruit slices using the *Agrobacterium*-mediated method. GUS activity and GUS staining of these infiltrated banana fruit slices were analyzed until 72 h after infiltration. The PB1121 empty vector was used as a positive control. These assays were repeated at least three times with similar results.

### 4.10. Statistical Analyses

A Student’s *t*-test was used for statistical analysis. The experimental results were expressed as the mean ± standard deviation (SD). Statistical significance was expressed by * *p* values < 0.05 and high statistical significance was expressed by ** *p* values < 0.01.

## 5. Conclusions

We identified 164 *MaWRKYs* based on version 2 of the *M*. *acuminata* genome and 162 *MaWRKYs* were located on 11 different chromosomes. All 164 *MaWRKYs* were classified into three groups (I, II, and III), and group II was classified into five sub-groups (IIa, IIb, IIc, IId, and IIe). The expression profiles of *MaWRKYs* were analyzed during different stages of fruit development and ripening and under different stressors. We found that many *MaWRKYs* were involved in banana fruit development and ripening stages; 10 *MaWRKYs* responded to drought, cold, and salt stress at the same time. *MaWRKY*s were systematically downregulated after inoculation with Foc4 TR4. *MaWRKY21* directly bound to the W-box of the *MaICS* promoter to decrease *MaICS* transcription and, in turn, enzyme activity; this is the first report of this in banana.

## Data Availability

The data presented in this study are available in the article and its Supplementary Materials.

## Supplementary Materials

The following are available online at https://www.mdpi.com/article/10.3390/plants11050662/s1; Table S1: The molecular weight and isoelectric points of the *MaWRKYs*. Table S2: Gene list of segmental and tandem duplication. Table S3: Sample of tissues and stage/treatment used for RNA-Seq. Table S4: The expression data of *MaWRKY**s* in banana fruit development and ripening stages. Table S5: The expression data (log2 Ratio Cold/Control) of the *MaWRKY**s* after cold treatment. Table S6: The expression data (log2 Ratio Osmotic/Control) of the *MaWRKY**s* after osmotic treatment. Table S7: The expression data (log2Ratio Salt/Control) of the *MaWRKY**s* after salt treatment. Table S8: The expression data (log2 Ratio 2 DPI/0 DPI) of the *MaWRKY**s* after inoculated Foc TR4. Table S9: PCR primers for expression analyses of *MaWRKYs*. Table S10: The characteristics of linked proteins with *MaWRKY21*. Table S11: Comparison with the number of genes in previous articles. The red font is the different gene in the two versions.
